# Cultivation-Based Quantification and Identification of Bacteria at Two Hygienic Key Sides of Domestic Washing Machines

**DOI:** 10.3390/microorganisms9050905

**Published:** 2021-04-23

**Authors:** Susanne Jacksch, Huzefa Zohra, Mirko Weide, Sylvia Schnell, Markus Egert

**Affiliations:** 1 Faculty of Medical and Life Sciences, Institute of Precision Medicine, Microbiology and Hygiene Group, Furtwangen University, 78054 Villingen-Schwenningen, Germany; Susanne.Jacksch@hs-furtwangen.de (S.J.); zohra.huzefa@gmail.com (H.Z.); 2 Research Centre for BioSystems, Land Use, and Nutrition (IFZ), Institute of Applied Microbiology, Justus-Liebig-University Giessen, 35392 Giessen, Germany; sylvia.schnell@umwelt.uni-giessen.de; 3 International Research & Development–Laundry & Home Care, Henkel AG & Co. KGaA, 40191 Düsseldorf, Germany; mirko.weide@henkel.com

**Keywords:** washing machine, bacteria, hygiene, MALDI biotyping

## Abstract

Detergent drawer and door seal represent important sites for microbial life in domestic washing machines. Interestingly, quantitative data on the microbial contamination of these sites is scarce. Here, 10 domestic washing machines were swab-sampled for subsequent bacterial cultivation at four different sampling sites: detergent drawer and detergent drawer chamber, as well as the top and bottom part of the rubber door seal. The average bacterial load over all washing machines and sites was 2.1 ± 1.0 × 10^4^ CFU cm^−2^ (average number of colony forming units ± standard error of the mean (SEM)). The top part of the door seal showed the lowest contamination (11.1 ± 9.2 × 10^1^ CFU cm^−2^), probably due to less humidity. Out of 212 isolates, 178 (84%) were identified on the genus level, and 118 (56%) on the species level using matrix-assisted laser desorption/ionization (MALDI) Biotyping, resulting in 29 genera and 40 identified species across all machines. The predominant bacterial genera were *Staphylococcus* and *Micrococcus*, which were found at all sites. 22 out of 40 species were classified as opportunistic pathogens, emphasizing the need for regular cleaning of the investigated sites.

## 1. Introduction

Representing wet, warm, and nutrient-rich environments, many sites of domestic washing machines offer ideal living conditions for microorganisms, such as bacteria and fungi [1,2]. Microbial contamination of washing machines might cause unaesthetic staining as well as malodor formation [3,4]. In addition, microbial biofilms might serve as reservoirs for (potentially) pathogenic microorganisms that might contaminate the laundry and thereby pose a health threat for susceptible persons [5,6].

Various studies have shown that washing machines are colonized by a considerable diversity of microbes, often capable of forming biofilms [3,7,8,9,10]. For instance, Nix and co- workers [10] investigated pro- and eukaryotic microorganisms on the rubber door seal and the detergent drawer using 16S rRNA gene and ITS1 region pyrosequencing. They identified taxa affiliated with *Proteobacteria* as the main bacterial representatives and *Basidiomycota* and *Ascomycota* representatives as the main fungal colonizers [10].

Regarding bacteria, washing machines are indeed mainly populated by the phyla *Proteobacteria, Actinobacteria, Firmicutes*, and *Bacteroidetes [7,9,10].* They largely enter the machine via soiled clothing, tap water, and maybe also air [2,4]. In a recent molecular study on the bacterial community of domestic washing machines, we identified the detergent drawer as the site with the highest bacterial diversity and the door seal as the site with highest relative abundance of malodor forming *Moraxella osloensis* species. Per site, 30–60% of the relatively most abundant sequence types were closely related to potentially pathogenic bacteria, such as *Brevundimonas vesicularis* or *Pseudomonas aeruginosa* inside the detergent drawer, and *Moraxella osloensis* or *Acinetobacter parvus* inside the door seal [9]. In a startling study, an antibiotic resistant *Klebsiella oxytoca* strain was recently isolated from biofilms of the detergent drawer and door seal of a domestic washing machine used for the woollen laundry of a paediatric hospital ward, from which it probably had colonized newborns [11].

Interestingly, quantitative data on the microbial contamination of different sites of domestic washing machines is scarce. To increase knowledge in this field, we aerobically cultivated and quantified bacteria from two sites of the detergent drawer and the door seal region of 10 domestic washing machines, each, and identified representative isolates by matrix-assisted laser desorption/ionization (MALDI) Biotyping.

## 2. Materials and Methods

### 2.1. Washing Machine Sampling

Swab samples were taken from 10 domestic (home-owned), front loading washing machines in the greater area of Villingen-Schwenningen, Germany, between April and June 2020. Each machine was sampled at the detergent drawer, the detergent drawer chamber, and the top and bottom parts of the rubber door seal. All tested machines were provided voluntarily by their owners. Similar sites of each machine were swapped with sterile cotton swaps (Deltalab, Rubí, Spain) pre-moistened in sterile physiological (0.9%) saline solution. The sampling area was ~42 cm^2^ for the detergent drawer, ~28 cm^2^ for the detergent chamber, and ~45 cm^2^ for the upper and lower parts of the rubber door seal, respectively. After sampling, the swab heads were transferred to a sterile reaction tube containing 2 mL of sterile physiological saline solution. All samples were processed within 1 h after sampling.

### 2.2. Colony Counting

Colony counting was performed as previously described in König et al. [12] and Egert et al. [13] with minor modifications. Swab heads were vortexed for 1 min at maximum speed. After serial decimal dilution up to 10^−6^ with sterile physiological saline solution, 100 µL of each dilution were spread in duplicates on tryptic soy agar plates (TSA; Carl Roth Karlsruhe, Germany) and incubated under aerobic conditions for 48 h at 37 °C. Subsequently, colonies in the range of 3 to 300 colonies were counted, averaged, and used for the calculation of microbial loads per cm^2^ of sample area.

One representative of each colony morphotype (differing in size, color, and/or colony morphology) per sample was picked with a sterile inoculation loop, re-streaked on TSA, and incubated aerobically at 37 °C. After control for purity, a colony from each morphotype was selected, dissolved in 300 µL of MALDI water (Honeywell, Offenbach, Germany), and stored at −80 °C for subsequent identification by MALDI Biotyping.

### 2.3. Identification of Isolates by MALDI Biotyping

The obtained isolates were identified with a MALDI Biotyper Microflex system (Bruker Daltonics, Bremen, Germany) according to the manufacturer’s instructions. The protein extraction method was applied using ethanol/formic acid sample preparation [14]. 1 µL of the respective protein extract of each isolated colony was added to a spot on the Biotyper steel target plate. After air drying, the samples were overlayed with 1 μL MALDI-matrix solution (alpha-Cyano-4-hydroxycinnamic acid, Bruker Daltonics, Bremen, Germany). After further air drying, the samples were analyzed. The obtained mass spectra were compared against the internal MALDI Biotyper reference libraries: MBT Compass Library, revision F, v. 9, containing 8468 main spectra (MSPs); MBT Filamentous Fungi Library (revision No. 2, containing 468 MSPs); MBT Security Related Library (SR Library, revision No. 1; containing 104 MSPs). Matches with the respective spectra in the databases were displayed as scores ranging from 0.0 to 3.0. Scores ≥ 1.7 indicated a secure genus identification and scores ≥ 2.0 a secure genus and probable species identification [15].

### 2.4. Statistical Analyses

The statistical analysis was performed using R (v. 3.6.1) [16] and R Studio (version 1.2.1335) [17] with the packages ggplot2 (v. 3.2.1) [18], reshape2 (v. 1.4.3) [19], and scales (v. 1.0.0) [20]. Non-parametric tests (Kruskal–Wallis rank sum test followed by Wilcoxon–Mann–Whitney post hoc tests) were used to check for statistical significance between the colony counts of the four sampling sites. *p*-values < 0.05 were considered as statistically significant.

## 3. Results and Discussion

### 3.1. Colony counts at the Different Sampling Sites

**Figure 1:**
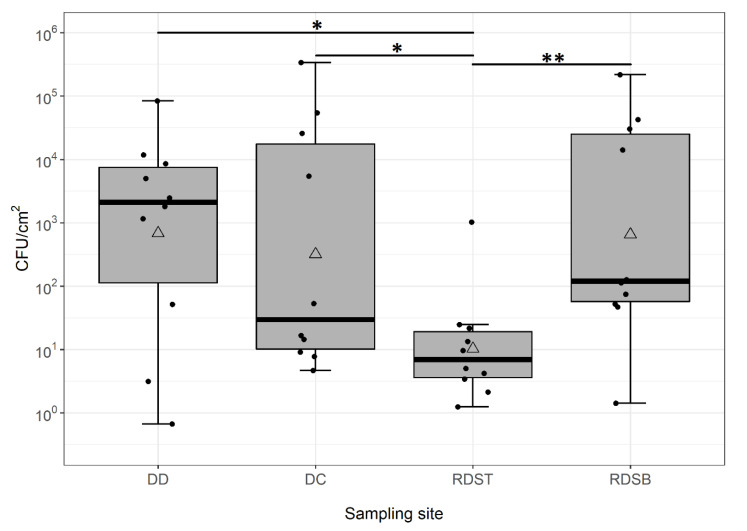
Box-whisker plots of aerobic colony counts per cm^2^ from 4 sampling sites of 10 domestic washing machines. Each box represents the 25% and 75% percentiles. Bold horizontal lines represent medians. Mean values are displayed as triangles. Whiskers above and below the boxes indicate the lowest and highest microbial counts that were not classified as outliers. Black points represent single data points per site. The different sampling sites are detergent drawer (DD), detergent drawer chamber (DC), top part of rubber door seal (RDST), and bottom part of rubber door seal (RDSB) (*n* = 10 for each sampling site). Results of Wilcoxon–Mann–Whitney post hoc tests are discussed in the text; significance levels are indicated by asterisks (* *p* < 0.05; ** *p* < 0.01).

All investigated samples showed microbial growth. Microbial loads spanned five orders of magnitude (Figure 1). The average colony count over all samples was 2.1 ± 1.0 × 10^4^ colony-forming units (CFU) cm^−2^ (average ± standard error of the mean (SEM)). The sampling site with the lowest cell numbers was the top part of the rubber door seal (RDST, 11.1 ± 9.2 × 10^1^ CFU cm^−2^), probably because water quickly drains off from here. Accumulation of (antimicrobial) detergent residues might be an additional reason.

Detergent drawer (DD), detergent drawer chamber (DC), and the bottom part of the rubber door seal (RDSB) showed similar values, with 1.1 ± 0.74 × 10^4^ CFU cm^−2^, 4.2 ± 3.0 × 10^4^ CFU cm^−2^, and 3.1 ± 1.9 × 10^4^ CFU cm^−2^, respectively. Statistical analysis by Kruskal–Wallis rank sum test proved a significant difference when comparing the colony counts of all sampling sites (*p* = 0.029, Figure 1). Subsequent pair-wise Wilcoxon–Mann–Whitney post hoc tests indicated differences between the top part of the rubber seal and its bottom part (*p* = 0.007), as well as the detergent drawer (*p* = 0.021) and the detergent drawer chamber (*p* = 0.045). Clearly, due to the large variability of the colony counts, studies with larger sample sizes are needed to substantiate these findings.

Interestingly, little is known about the microbial load of different sites inside domestic washing machines [21]. To the best of our knowledge, only Stapelton and colleagues [21] have previously reported the microbial loads of different sampling sites, albeit only for four domestic washing machines. While our data match their results for the rubber doors seal quite well (~ 10^3^ to 10^4^ cm^−2^), they also suggest the detergent drawer region as being significantly more contaminated than reported by them (~ 10^−1^ to 10^3^ cm^−2^). Clearly, also from a quantitative point for view, the detergent drawer region is an important site for washing machine hygiene and, thus, probably also laundry hygiene.

### 3.2. Identification of Microbial Isolates

212 microbial isolates stemming from the 40 washing machine samples were analyzed by MALDI Biotyping. Genus-level identification scores (≥1.7) were determined for 178 isolates (84%), while 34 isolates (16%) could not be identified. 118 isolates (56%) were probably identified on species level (score ≥2.0). In total, 29 genera and 40 species were found (Table 1).

Standard cultivation techniques are limited, as they only detect cultivable microorganisms and thus discriminate against the vast majority of microorganisms on earth [24]. Therefore, we particularly compared the results obtained here with data from previous molecular studies, in particular, a recent one conducted by us with machines from the same region [9].

In accordance with previous molecular studies [7,9,10], *Proteobacteria* (29%), *Actinobacteria* (27%), *Firmicutes* (26%), and *Bacteroidetes* (0.5%) also represented the most abundant phyla here. In accordance with the relatively most abundant species found in our previous molecular study [9], *Pseudomonas oleovorans*, *Acinetobacter parvus*, and *Moraxella osloensis* were also detected here by cultivation in the door seal, while *Rhizobium radiobacter* was detected in the detergent drawer (Table 1) [9].

Many of the identified species represent environmental bacteria, typically found in water habitats or the human body, such as skin-associated bacteria. In addition, some of the identified species are well-known biofilm formers, such as *Staphylococcus epidermidis*, *Micrococcus luteus*, *Bacillus cereus*, and *Pseudomonas sp.* [2,4,7,9,10,25,26,27,28].

To estimate their pathogenic potential, the identified bacterial species were classified into biosafety risk groups (RG) [22,23]. More than 50% (22 of 40 species) were affiliated with RG 2 organisms, i.e., representing a potential health risk, especially for immunocompromised patients, pregnant women, or elderly persons [15]. 15 out of 21 identified RG 2 bacteria were found in the detergent drawer compartment (DD and DC), and 13 out of 21 RG 2 bacteria on the entire rubber door seal.

By far, micrococci and staphylococci were the most frequently isolated genera, which is in contrast to the different molecular studies mentioned here [9,10] and might represent a cultivation bias. Micrococci and staphylococci represent ubiquitous microorganisms that are often isolated from the skin and mucous membranes of humans and animals, but also from air and water. They grow fast under a broad range of cultivation conditions [29,30,31]. However, they also have the ability of dormancy and might therefore well resist the dramatically changing environmental conditions inside washing machines [32,33]. The frequent detection of (non-pathogenic) micrococci on the rubber door seals might be due to the fact that these parts are more frequently touched by human hands than the other parts investigated here.

Staphylococci such as *S. epidermidis*, *S. lugdunensis*, *S. saprophyticus*, and *S. haemolyticus* possess a pathogenic potential, and may also play a role in the horizontal gene transfer of antibiotic resistance genes [34,35,36]. The presence and transmission of such resistance genes throughout washing machines have already been confirmed for β-lactamase [37]. β-lactamase-producing *Klebsiella oxytoca* and *Klebsiella pneumoniae* species have also been isolated from washing machines before [11,38]. It can be speculated that these bacteria can be transferred to other surfaces, e.g., via bioaerosols [34,35,36]. Notably, *Klebsiella oxytoca* was also found in our study; however, without knowing its resistance pattern. Clearly, the interaction between the chemistry used for cleaning and disinfection and the selection of (antibiotic) resistant microbial species is an important topic in laundry and household hygiene [39,40].

Besides bacteria, a few eukaryotic species were also isolated with the used cultivation conditions, all affiliated with *Ascomycota* (1 %). The most abundant genus was *Aspergillus*. *Aspergillus sp*. are saprophytic fungi and can recycle organic debris. *A. fumigatus* is a prevalent airborne fungal pathogen that can cause severe infections in immunocompromised people [41].

## 4. Conclusions

Despite its small sample size, our study clearly shows that both the detergent drawers and bottom door seals of domestic washing machines are significantly contaminated with cultivable bacteria, including significant shares of potentially pathogenic ones. Maximum loads can exceed 10^5^ CFU per cm^2^. For the sake of machine and laundry hygiene, both parts should be cleaned regularly. Markedly lower CFU counts from the top part of the door seal underline the importance of water for the microbial contamination of washing machines. When not in use, machines should be left open to dry out. Bacterial species identified here and in molecular studies as quantitatively important for the washing machine microbiota represent test organisms with high practical relevance for antimicrobial efficacy testing.

## Figures and Tables

**Table 1:** Number of microbial isolates obtained from 10 domestic washing machines and identified by matrix-assisted laser desorption/ionization (MALDI) Biotyping with identification scores ≥1.7 (genus level; *n*= 178) and ≥2.0 (species level; *n* = 118) across the four different sampling sites (DD = detergent drawer; DC = detergent drawer chamber; RDST = top part of rubber door seal; RDSB= bottom part of rubber door seal). Species categorized as risk group 2 (based on the German Rules for Biological Agents #446 [22] and #460 [23]) are marked with an asterisk. Species detected here which have been previously identified in (9) as one of the ten relatively most abundant species from door seals and detergent drawers, respectively, are written in bold.

Phylum	Class	Order	Family	Genus	Species	DD	DC	RDST	RDSB
**Actino-bacteria**	Actinobacteria	Actinomycetales	Dermacoccaceae	*Dermacoccus*	*Dermacoccus sp.*	-	-	1	-
					*Dermacoccus nishinomiyaensis*	-	-	-	1
		Corynebacteriales	Corynebacteriaceae	*Coryne-bacterium*	*Corynebacterium sp.*	-	2	1	-
					*Corynebacterium lipophiloflavum*	-	-	1	-
		Micrococcales	Brevibacteriaceae	*Brevibacterium*	*Brevibacterium celere*	-	1	-	-
			Dermatophilaceae	*Arsenicicoccus*	*Arsenicicoccus bolidensis*	1	-	-	-
			Micrococcaceae	*Kocuria*	*Kocuria sp.*	-	1	1	3
					*Kocuria rhizophila*	-	1	2	1
				*Micrococcus*	*Micrococcus sp.*	-		10	3
					*Micrococcus luteus*	2	2	13	11
**Bactero-idetes**	Sphingobacteriia	Sphingomonadales	Sphingobacteriaceae	*Sphingo-bacterium*	Sphingobacterium spiritivorum *	1	-	-	-
**Firmicutes**	Bacilli	Bacillales	Bacillaceae	*Bacillus*	*Bacillus sp.*	5		2	2
					Bacillus cereus *	1	3	1	1
					*Bacillus licheniformis*	-	1	-	-
					*Bacillus megaterium*	1	-	1	1
				*Lysinibacillus*	*Lysinibacillus sp.*	-	-	-	1
			Paenibacillaceae	*Paenibacillus*	*Paenibacillus residui*	-	-	-	1
			Planococcaceae	*Solibacillus*	*Solibacillus sp.*	1	-	-	-
			Staphylococcaceae	*Staphylococcus*	*Staphylococcus sp.*	2	5	5	4
					*Staphylococcus capitis*	-	-	1	
					Staphylococcus epidermidis *	-	1	-	3
					Staphylococcus haemolyticus *	2	-	-	-
					Staphylococcus hominis *	-	1	-	1
					Staphylococcus lugdunensis *	1	-	-	-
					*Staphylococcus saprophyticus*	-	-	-	1
					*Staphylococcus warneri*	1	2	3	
**Proteo-bacteria**	Alphaproteobacteria	Rhizobiales	Rhizobiaceae	*Rhizobium*	*Rhizobium radiobacter*	-	1	-	3
		Rhodospirillales	Acetobacteraceae	*Roseomonas*	Roseomonas mucosa *	-	2	-	-
		Sphingomonadales	Sphingomonadaceae	*Sphingomonas*	*Sphingomonas sp.*	1	-	-	-
					Sphingomonas paucimobilis *	1	-	-	-
					*Sphingomonas pseudosanguinis*	1	-	-	-
	Betaproteobacteria	Burkholderiales	Alcaligenaceae	*Achromobacter*	*Achromobacter sp.*	-	-	1	-
					Achromobacter mucicolens *	-	1	-	-
			Comamonadaceae	*Delftia*	*Delftia acidovorans*	2	-	-	-
	Gammaproteobacteria	Aeromonadales	Aeromon-adaceae	*Aeromonas*	Aeromonas caviae *	-	-	-	1
**Phylum**	Class	Order	Family	Genus	Species	DD	DC	RDST	RDSB
**Proteo-bacteria**	Gammaproteobacteria	Alteromonadales	Alteromonadaceae	*Alishewanella*	*Alishewanella sp.*	1	-	-	-
			Shewanellaceae	*Shewanella*	Shewanella putrefaciens *	-	-	-	1
		Enterobacteriales	Enterobacteriaceae	*Citrobacter*	Citrobacter freundii *	-	-	-	1
					Citrobacter gillenii *	-	-	-	2
				*Klebsiella*	Klebsiella oxytoca *	-	1	-	2
				*Pantoea*	Pantoea agglomerans *	1	-	-	-
		Pseudomonadales	Moraxellaceae	*Acinetobacter*	Acinetobacter johnsonii *	1	-	-	-
					Acinetobacter lwoffii *	1	-	-	1
					Acinetobacter parvus *	-	-	-	1
					Acinetobacter ursingii *	-	-	-	5
				*Moraxella*	*Moraxella sp.*	-	1	1	-
					Moraxella osloensis *	1	1	-	2
			Pseudomonadaceae	*Pseudomonas*	*Pseudomonas sp.*	4	-	-	-
					*Pseudomonas alcaliphila*	5	1	-	-
					*Pseudomonas oleovorans*	2	3	-	1
					*Pseudomonas stutzeri*	1	2	-	
		Xanthomonadales	Xanthomonadaceae	*Stenotro-phomonas*	Stenotrophomonas maltophilia *	1	1	1	1
**Asco-mycota**	Eurotiomycetes	Eurotiales	Trichocomaceae	*Aspergillus*	*Aspergillus sp.*	-	-	-	1
					Aspergillus fumigatus *	1	-	-	-
	Saccharomycetes	Saccharomycetales	Debaryomycetaceae	*Candida*	*Candida sp.*	-	1	-	-

## Data Availability

Not applicable.

## References

[1] Babič M.N., Zalar P., Ženko B., Schroers H.-J., Džeroski S., Gunde-Cimerman N. (2015). *Candida* and *Fusarium* species known as opportunistic human pathogens from customer-accessible parts of residential washing machines. Fungal Biol..

[2] Babič M.N., Gostinčar C., Gunde-Cimerman N. (2020). Microorganisms populating the water-related indoor biome. Appl. Microbiol. Biotechnol..

[3] Egert M. (2017). The BE-Microbiome-Communities with Relevance for Laundry and Home Care. SOFW J..

[4] Munk S., Johansen C., Stahnke L.H., Adler-Nissen J. (2001). Microbial survival and odor in laundry. J. Surfact. Deterg..

[5] Bloomfield S.F., Exner M., Goroncy-Bermes P., Hartemann P., Heeg P., Ilschner C. (2015). Lesser-known or hidden reservoirs of infection and implications for adequate prevention strategies: Where to look and what to look for. GMS Hyg. Infect. Control.

[6] Gibson L.L., Rose J.B., Haas C.N. (1999). Use of quantitative microbial risk assessment for evaluation of the benefits of laundry sanitation. Am. J. Infect Control..

[7] Callewaert C., van Nevel S., Kerckhof F.M., Granitsiotis M.S., Boon N. (2015). Bacterial Exchange in Household Washing Machines. Front. Microbiol..

[8] Gattlen J., Amberg C., Zinn M., Mauclaire L. (2010). Biofilms isolated from washing machines from three continents and their tolerance to a standard detergent. Biofouling.

[9] Jacksch S., Kaiser D., Weis S., Weide M., Rtering S., Schnell S., Egert M. (2020). Influence of Sampling Site and other Environmental Factors on the Bacterial Community Composition of Domestic Washing Machines. Microorganisms.

[10] Nix I.D., Frontzek A., Bockmühl D.P. (2015). Characterization of Microbial Communities in Household Washing Machines. Tenside Surfactants Deterg..

[11] Schmithausen R.M., Sib E., Exner M., Hack S., Rösing C., Ciorba P., Bierbaum G., Savin M., Bloomfield S.F., Kaase M. (2019). The Washing Machine as a Reservoir for Transmission of Extended-Spectrum-Beta-Lactamase (CTX-M-15)-Producing *Klebsiella oxytoca* ST201 to Newborns. Appl. Environ. Microbiol..

[12] König C., Tauchnitz S., Kunzelmann H., Horn C., Blessing F., Kohl M., Egert M. (2017). Quantification and identification of aerobic bacteria in holy water samples from a German environment. J. Water Health.

[13] Egert M., Späth K., Weik K., Kunzelmann H., Horn C., Kohl M., Blessing F. (2015). Bacteria on smartphone touchscreens in a German university setting and evaluation of two popular cleaning methods using commercially available cleaning products. Folia Microbiol..

[14] Wang J., Wang H., Cai K., Yu P., Liu Y., Zhao G., Chen R., Xu R., Yu M. (2021). Evaluation of three sample preparation methods for the identification of clinical strains by using two MALDI-TOF MS systems. J. Mass Spectrom..

[15] Fritz B., Jenner A., Wahl S., Lappe C., Zehender A., Horn C., Blessing F., Kohl M., Ziemssen F., Egert M. (2018). A view to a kill?—Ambient bacterial load of frames and lenses of spectacles and evaluation of different cleaning methods. PLoS ONE.

[16] R Core Team (2018). R: A language and environment for statistical computing. Vienna, Austria: R Foundation for Statistical Computing. https://cran.r-project.org/.

[17] RStudio Team (2021). RStudio: Integrated Development for R. R Studio.

[18] Wickham H., Sievert C. (2016). ggplot2: Elegant Graphics for Data Analysis.

[19] Wickham H. (2007). Reshaping Data with the reshape Package. J. Stat. Soft..

[20] Wickham H., Seidel D. (2018). scales: Scale Functions for Visualization. https://www.rdocumentation.org/packages/scales.

[21] Stapleton K., Hill K., Day K., Perry J.D., Dean J.R. (2013). The potential impact of washing machines on laundry malodour generation. Lett Appl Microbiol..

[22] BAuA—German Federal Institute for Occupational Safety and Health (2015). Technical Rule for biological agents (TRBA) # 466: Classification of Prokaryotes (Bacteria and Archaea) into Risk Groups. https://www.baua.de/DE/Angebote/Rechtstexte-und-Technische-Regeln/Regelwerk/TRBA/TRBA-466.html.

[23] BAuA—German Federal Institute for Occupational Safety and Health (2016). Technical Rule for Biological Agents (TRBA) #460: Classification of Fungi into Risk Groups. https://www.baua.de/DE/Angebote/Rechtstexte-und-Technische-Regeln/Regelwerk/TRBA/TRBA-460.html..

[24] Lloyd K.G., Ladau J., Steen A.D., Yin J., Crosby L. (2018). Phylogenetically Novel Uncultured Microbial Cells Dominate Earth Microbiomes. mSystems.

[25] Otto M. (2008). Staphylococcal biofilms. Curr. Top Microbiol. Immunol..

[26] Vlamakis H., Chai Y., Beauregard P., Losick R., Kolter R. (2013). Sticking together: Building a biofilm the Bacillus subtilis way. Nat. Rev. Microbiol..

[27] Matsuura K., Asano Y., Yamada A., Naruse K. (2013). Detection of *Micrococcus luteus* biofilm formation in microfluidic environments by pH measurement using an ion-sensitive field-effect transistor. Sensors.

[28] Mann E.E., Wozniak D.J. (2012). *Pseudomonas* biofilm matrix composition and niche biology. FEMS Microbiol. Rev..

[29] Chiller K., Selkin B.A., Murakawa G.J. (2001). Skin microflora and bacterial infections of the skin. J. Investig. Dermatol. Symp. Proc..

[30] Götz F., Bannerman T., Schleifer K.-H., Dworkin M., Falkowm S., Rosenbergm E., Schleifer K.-H., Stackebrandt E. (2006). The Genera *Staphylococcus* and *Macrococcus*. The Prokaryotes.

[31] Fang Z., Ouyang Z., Zheng H., Wang X., Hu L. (2007). Culturable airborne bacteria in outdoor environments in Beijing, China. Microb. Ecol..

[32] Kaprelyants A.S., Kell D.B. (1993). Dormancy in Stationary-Phase Cultures of *Micrococcus luteus*: Flow Cytometric Analysis of Starvation and Resuscitation. Appl. Environ. Microbiol..

[33] Cerca F., França Â., Pérez-Cabezas B., Carvalhais V., Ribeiro A., Azeredo J., Pier G., Cerca N., Vilanova M. (2014). Dormant bacteria within *Staphylococcus epidermidis* biofilms have low inflammatory properties and maintain tolerance to vancomycin and penicillin after entering planktonic growth. J. Med. Microbiol..

[34] Madsen A.M., Moslehi-Jenabian S., Islam M.Z., Frankel M., Spilak M., Frederiksen M.W. (2018). Concentrations of *Staphylococcus* species in indoor air as associated with other bacteria, season, relative humidity, air change rate, and *S. aureus*-positive occupants. Environ. Res.

[35] Kooken J.M., Fox K.F., Fox A. (2012). Characterization of *Micrococcus* strains isolated from indoor air. Mol. Cell Probes..

[36] Brandl H., Fricker-Feer C., Ziegler D., Mandal J., Stephan R., Lehner A. (2014). Distribution and identification of culturable airborne microorganisms in a Swiss milk processing facility. J. Dairy Sci..

[37] Rehberg L., Frontzek A., Melhus Å., Bockmühl D.P. (2017). Prevalence of β-lactamase genes in domestic washing machines and dishwashers and the impact of laundering processes on antibiotic-resistant bacteria. J. Appl. Microbiol..

[38] Boonstra M.B., Spijkerman D.C.M., Voor In ’t Holt A.F., van der Laan R.J., Bode L.G.M., van Vianen W. (2020). An outbreak of ST307 extended-spectrum beta-lactamase (ESBL)-producing *Klebsiella pneumoniae* in a rehabilitation center: An unusual source and route of transmission. Infect. Control. Hosp. Epidemiol..

[39] Bockmühl D.P. (2017). Laundry hygiene-how to get more than clean. J. Appl. Microbiol..

[40] Velazquez S., Griffiths W., Dietz L., Horve P., Nunez S., Hu J., Shen J., Fretz M., Bi C., Xu Y. (2019). From one species to another: A review on the interaction between chemistry and microbiology in relation to cleaning in the built environment. Indoor Air..

[41] Latgé J.P. (1999). *Aspergillus fumigatus* and aspergillosis. Clin. Microbiol. Rev..

